# Possible recurrence of neuromuscular block following balloon dilation for bronchial stenosis in a young patient after bilateral lung transplantation: A case report

**DOI:** 10.1097/MD.0000000000042614

**Published:** 2025-05-23

**Authors:** Taiga Ishihara, Nobuko Ito, Kanji Uchida

**Affiliations:** a Department of Anesthesiology and Pain Relief Center, University of Tokyo Hospital, Tokyo, Japan.

**Keywords:** anaphylaxis, case report, neuromuscular blockade, recurarization, rocuronium

## Abstract

**Rationale:**

Sugammadex is a synthetic cyclodextrin derivative that reverses the effects of nondepolarizing neuromuscular blockers through selective encapsulation. Residual neuromuscular blockade (NMB) after extubation can lead to adverse events such as hypoxia and cardiac arrest. We encountered a case of residual NMB, despite the appropriate use of NMB monitoring and sugammadex, as recommended by the American Society of Anesthesiologists.

**Patient concerns:**

A 27-year-old female underwent balloon dilatation under general anesthesia for left bronchial stenosis after lung transplantation. Rocuronium (50 mg) was administered for intubation, with 10 mg intermittently administered to maintain a train-of-four (TOF) count of <2. At the conclusion of the procedure, sugammadex (150 mg) was administered at a TOF count of 3. The patient was extubated with a TOF ratio of 100%. Fifteen minutes after transfer to the ward and 30 minutes after sugammadex administration, the patient developed respiratory distress and erythema on her trunk and upper limbs, exhibiting possible symptoms of anaphylaxis. The emergency team administered adrenaline via intramuscular injection. Despite this, the respiratory symptoms worsened.

**Diagnoses:**

Difficulty in breathing was possibly due to recurrent NMB caused by residual muscle relaxants that were not fully reversed.

**Interventions:**

The patient was reintubated and transferred to the intensive care unit. Bronchoscopy performed after reintubation did not reveal signs of anaphylactic airway swelling.

**Outcomes:**

The patient’s respiratory condition improved, and she was extubated the following morning. After extubation, she recalled being unable to breathe or move during the follow-up bronchoscopy. Plasma histamine and serum tryptase levels were measured 2 and 24 hours after the suspected anaphylactic episode, showing no increase in the levels.

**Lessons:**

This case highlights that neuromuscular monitoring and adherence to sugammadex dosing guidelines do not entirely eliminate the risk of recurarization, which may present with anaphylaxis-like symptoms, complicating the diagnosis.

## 1. Introduction

Sugammadex is a synthetic cyclodextrin derivative that reverses the effects of nondepolarizing neuromuscular blockers through selective encapsulation. First approved in 2010, sugammadex has since been widely used in Japan. Residual neuromuscular blockade (NMB) after extubation may lead to adverse events, including hypoxia due to decreased respiratory function and cardiac arrest.^[1–4]^ Therefore, the American Society of Anesthesiologists (ASA) recommends quantitative monitoring, such as acceleromyography and electromyography, rather than clinical assessment alone. Additionally, the ASA practice guidelines recommend confirming a train-of-four (TOF) ratio ≥ 0.9 before extubation.^[5]^ However, we encountered a case of recurarization despite the appropriate use of NMB monitoring and sugammadex. Moreover, recurrence occurred in the ward, outside the observation scope of the anesthesiology team, and was initially suspected and treated as anaphylaxis.

## 2. Case presentation

A case involving a 27-year-old female (height, 156 cm; weight, 56 kg) underwent balloon dilation for bronchial stenosis. She had been diagnosed with idiopathic pulmonary arterial hypertension 12 years prior. Due to worsening pulmonary hypertension (right ventricular systolic pressure: 90 mm Hg), she underwent bilateral lung transplantation. Posttransplant, she received enteral mycophenolate mofetil and intravenous hydrate and methylprednisolone, with careful monitoring of tacrolimus trough levels.

On postoperative day (POD) 3 after transplantation, she was weaned off extracorporeal membrane oxygenation, her chest wall was closed, and she was extubated on POD 6. A thrombus in the left lower pulmonary vein necessitated thrombectomy and lobectomy on POD 13. She was transferred to the general ward on POD 20, after transplantation. Computed tomography revealed left bronchial stenosis and she required 0.5 L/min. oxygen during exercise. On day 69 posttransplant, she underwent balloon dilation under general anesthesia.

Anesthesia was induced with intravenous fentanyl (100 µg) and propofol (150 mg). After confirming loss of verbal response and eyelash reflex, an acceleromyographic monitor was calibrated, and rocuronium (50 mg) was administered. TOF monitoring (AF-101P, Nihon-Kohden Corporation, Tokyo, Japan) was performed on the adductor pollicis muscle every 15 seconds (current amplitude, 50 mA; pulse width, 200 s). Continuous propofol and remifentanil infusion was maintained, with additional rocuronium (10 mg) every 10 minutes when a TOF count of 2 was observed. Bispectral index values remained between 40 and 60, and esophageal temperature was maintained at 36.2 to 36.5°C.

Bronchoscopy revealed the membranous portion was compressed by granulation tissue from the prior left lower lobectomy. A 1,00,000-fold solution of adrenaline was applied to the stenotic areas by the surgeons. Balloon dilation was performed 3 times for 2 minutes each on the B3 and lingular branches. No active bleeding was observed, and the procedure was completed in 55 minutes. TOF count of 3 was confirmed and sugammadex (150 mg [equivalent to 2 mg/kg]) was administered. Upon awakening, the patient’s vital signs were stable and she was able to follow verbal commands with a tidal volume of 400 mL, end-tidal carbon dioxide of 46 mm Hg, and a respiratory rate of 12 breaths/min. A TOF ratio of 100% was confirmed, and the patient was extubated. She was able to converse without any issues, reported no pain or dyspnea, and was transferred to the general ward. The total duration of anesthesia was 1 hour 48 minutes with a cumulative rocuronium dose of 130 mg.

Fifteen minutes after returning to the ward and 30 minutes post-sugammadex administration, the patient developed progressive dyspnea and decreased consciousness. Stridor was noted as SpO_2_ dropped to 62%, accompanied by mottled erythema on the trunk and upper limbs, raising suspicion of anaphylaxis. Intramuscular adrenaline (0.5 mg) was administered. Bag-mask ventilation with 100% oxygen improved SpO_2_ rapidly. Chest radiography ruled out pneumothorax and atelectasis. However, spontaneous breathing remained unstable, and SpO_2_ rapidly declined when positive pressure ventilation ceases. Arterial blood gas analysis showed a partial carbon dioxide of 97.3 mm Hg, leading to incubation and ICU transfer for mechanical ventilation.

Repeat bronchoscopy revealed edema and narrowing of the left main bronchus and upper lobe branches but did not suggest airway edema due to anaphylaxis. Plasma histamine and serum tryptase levels were measured at 2 and 24 hours after possible anaphylactic episodes. The 2 and 24 hours measurements of plasma histamine level were 1.3 and 1.2 µg/L, and serum tryptase level was 1.01 and 1.56 µg/mL, respectively, which exhibited no increase in the levels of either. As the event was identified an hour after it occurred, the operators did not have the opportunity to measure plasma histamine and serum tryptase levels at the time of occurrence.

The patient’s respiratory condition improved and she was extubated the following morning after several follow-up bronchoscopies. In subsequent interviews, she recalled being unable to breathe and attempting to alert medical staff by fluttering her toes, which she had managed to move.

## 3. Discussion

We encountered a case of recurarization (i.e., recurrent NMB) despite NMB monitoring and administration of an adequate dose of sugammadex, as prescribed on the drug package insert. The ASA Task Force on NMB analyzed multiple studies and reported that when a TOF ratio ≥ 0.9, was confirmed before extubation, the incidence proportion of residual NMB (TOF ratio < 0.9) was 0.5%, whereas this proportion increased to 2.2% when not confirmed.^[5]^ According to the prescription information from Merke Sharp and Dohme Corp.,^[6]^ the incidence of recurarization after administering the recommended dose of sugammadex is <1%.

The emergency team considered several possible causes for the respiratory distress, including anaphylaxis, a reaction to residual fentanyl, bronchial narrowing due to edema, or bleeding from the bronchial dilation procedure. They noted mottled erythema on the patient’s trunk and upper limbs, treated her for anaphylaxis, and administered intramuscular adrenaline. International guidelines recommend that adrenaline should be administered as soon as signs of anaphylaxis appear.^[7]^

However, we believe that the respiratory distress symptoms were the result of recurarization rather than anaphylaxis for several reasons. First, the plasma histamine and serum tryptase levels did not increase at 2 and 24 hours, even after anaphylaxis was suspected. Secondly, the patient recalled being unable to move during bronchoscopy in the ICU, which strongly indicated the possibility of recurrence. In addition, in a systematic review of 23 cases of anaphylaxis to sugammadex, Zecic et al^[8]^ reported that in 87% of cases, the symptoms occurred very early, within 5 minutes after sugammadex administration, with the time to onset of symptoms ranging from 0 to 8 minutes, which is another reason why we considered our case unlikely to be an anaphylactic reaction. In our case, the patient experienced difficulty in breathing 30 minutes after sugammadex administration. Because the patient developed erythema, the possibility of an anaphylactic reaction(s) could not be completely ruled out. However, the emergence of a rash, which is indicative of anaphylaxis, can be considered as a stress response resulting from dyspnea. Therefore, it is highly likely that the breathing difficulty experienced by the patient was due to recurrent NMB caused by residual muscle relaxants that were not fully antagonized.

Because our patient underwent lung transplantation before the operation, she was administered immunosuppressants and steroids. Although a limited number of reports have addressed the relationship between rocuronium and tacrolimus, some immunosuppressive drugs can prolong the action of NMBs,^[9]^ which may have been the case with our patient.

In the present case, the TOF was 0 after each rocuronium administration. However, the patient spontaneously recovered to TOF > 2 or TOF > 3 within 10 minutes. As the surgeons asked for immobilization during the procedure, rocuronium (10 mg) was administered when TOF recovered to ≥2, which resulted in a considerably high total dose of rocuronium. However, we sometimes encounter patients who exhibit spontaneous recovery to TOF > 2 shortly after additional administration of rocuronium, and recurarization may occur even with the appropriate use of an NMB monitor and sugammadex. It has been reported that 3.57 mg of sugammadex (molecular weight, 2178 kDa) is required to encapsulate 1.0 mg of rocuronium (molecular weight, 610 kDa)^[10]^ and, theoretically, 150 mg of sugammadex may be insufficient for the reversal of 130 mg of rocuronium. As the concentration of free rocuronium in the central compartment decreases after sugammadex administration, rocuronium may be redistributed from the periphery to the central and effect-site compartments, potentially leading to recurarization.^[4]^ Continuous NMB monitoring and observation in the postanesthesia care unit should be considered when a higher dose of rocuronium is administered. If recurarization occurs outside the supervision of an anesthesiologist, respiratory distress may manifest as anaphylaxis-like symptoms, potentially complicating the diagnosis. In our case, the TOF was not measured in the ward, which may be a limitation.

## 4. Conclusion

Recurarization may occur even with appropriate NMB monitoring and sugammadex reversal. When administering higher doses of NMB for short surgical procedures, it is crucial to not only monitor neuromuscular function but also adjust the sugammadex dose based on the total NMB administered. Additionally, extended observation in the recovery room should be considered. Anesthesiologists should warn surgeons of the risk of recurarization in the ward. Without anesthesiologist supervision, proper diagnosis of recurarization may be challenging. Therefore, educating emergency teams and ward staff about NMB-related side effects is essential.

## Author contributions

**Conceptualization:** Taiga Ishihara, Nobuko Ito.

**Data curation:** Taiga Ishihara, Nobuko Ito.

**Investigation:** Taiga Ishihara, Nobuko Ito.

**Writing – original draft:** Taiga Ishihara.

**Writing – review & editing:** Nobuko Ito, Kanji Uchida.
